# Notes and Comments

**Published:** 1899-02

**Authors:** 


### Notes and Comments.[1]

Bur-Excavation.—Some one has said that if the bur is repeatedly dipped in oil of cloves while excavating, the pain will be much lessened. Such things are often said, and go unheeded because they are so simple, while many a time the simplest truths are the greatest truths. However, if there is any merit in the above, it is because of the lubrication of the bur, thereby lessening the friction, which lessens the heat that constant burring causes.—*Dental Weekly.*

Extraordinary Results.—The *Indiana Dental Journal* says that at a recent meeting of the Odontological Society of Indianapolis, Dr. Merit Wells reported a case where a child less than two years of age, while swinging a bottle up and down, struck one of the deciduous central incisors with such force that it was driven into the alveolar process so that the crown was almost totally obscured. He supposed that the accident would result in the loss of the deciduous tooth, and probably in death of the permanent tooth-germ. Strange to say, the deciduous tooth gradually regained its normal position with the pulp living, and the permanent central is now fully developed and in position.

Delicacy in Operating.—In writing upon this subject the editor of the *Practitioner* says if there be any one thing which a dentist should cultivate, it is delicacy and lightness of touch. Some dentists whom we have known go at their work like a miner with a pick-axe. They are rough, harsh, and their hand, whether with the excavator, the plugger, or engaged in adjusting the various appliances of our art, is ever heavy. Their arms always rest burdensomely upon the patient's head. Their finger-nails are continually digging into tender tissues, and there is a coarseness and clumsiness about their operations that marks an unpardonable heedlessness of the comfort of the patient. There are few things

[1] The assistant editor solicits contributions for this department,—new methods, new remedies and formulas, or any short practical note which may prove of value to the practitioner or student. Address 1338 Walnut Street, Philadelphia.

which so forcibly commend an operator to those under his care as tenderness, and even daintiness, in regard to their sensibilities. The engine-bur should be directed as if it were a sentient thing, and napkins should be used as though they were a spontaneous production.

---

A Method of repairing a Broken Facing.—In a paper read before the Northeastern Dental Association, Dr. George F. Harwood offered the following as his method of repairing broken facings in bridge-work: First drill through the backing for the pins of the new facing, cutting out the gold between, countersinking the backing on the palatal surface, forcing the pins apart and into the countersink by a pair of curved pliers, one jaw of which is fitted with a swivelled socket to receive a pellet of lead or shot to protect the facing, the other having a grooved and swivelled socket, wedge-shaped, to force the pins apart and into the countersink, while that part between the pins may be filled with a fine alloy from which the excess of mercury has been carefully expressed.

---

A Process for Nickel Plating.—The following process for nickel plating is given in the *American Druggist:* Boil in a copper vessel a saturated solution of zinc chloride and an equal quantity of water. While boiling add hydrochloric acid, drop by drop, until the precipitate at first thrown down is redissolved. Now add zinc in powder, until the bottom of the kettle is nearly covered with a precipitate of zinc. The bath is now ready for the addition of a salt of nickel, and you may use either the sulphate or the nitrate. Add it in sufficient quantity to give the bath a strong green color. The articles to be nickelled are now hung in the bath by means of a zinc wire, or a strip of sheet zinc, and a few pieces of the latter are thrown in along with them. Raise the heat to a strong boil and continue it for several minutes, or until the articles are covered with a bright coating of nickel. The articles should be thoroughly cleaned and free from grease before being put in the bath.

---

Borax Solution for Soldering.—Dr. J. T. Usher gives his method of making a solution in the *Dental Cosmos.* He says, "In soldering gold crowns I use a saturated aqueous solution of borax, made by filling a bottle with water and dropping into it a lump of borax. This is allowed to boil on top of my vulcanizer or elsewhere, and the water will take up a certain amount of the borax, leaving the residue undissolved. An ounce of this solution will last a busy man about a year. In using it the piece to be soldered is simply moistened where the solder is wanted to flow, and the solder will run like a flash, much easier than when the borax powder is used."

